# Multi-omics analyses of the gut microbiome, fecal metabolome, and multimodal brain MRI reveal the role of *Alistipes* and its related metabolites in major depressive disorder

**DOI:** 10.1017/S003329172510072X

**Published:** 2025-07-07

**Authors:** Siyu Liu, Yifei Li, Yu Shi, Zhonghao Rao, Yongqi Zhang, Yu Zhang, Ting Wang, Hui Kong, Shukun Zhu, Dao-min Zhu, Yongqiang Yu, Jiajia Zhu

**Affiliations:** 1 Department of Radiology, The First Affiliated Hospital of Anhui Medical University, Hefei, China; 2 Research Center of Clinical Medical Imaging, Anhui Province, Hefei, China; 3 Anhui Provincial Institute of Translational Medicine, Hefei, China; 4 Anhui Provincial Key Laboratory for Brain Bank Construction and Resource Utilization, Hefei, China; 5 Department of Sleep Disorders, Affiliated Psychological Hospital of Anhui Medical University, Hefei, China; 6 Anhui Mental Health Center, Hefei, China; 7 Hefei Fourth People’s Hospital, Hefei, China

**Keywords:** *Alistipes*, brain imaging, fecal metabolome, gut microbiota, major depressive disorder

## Abstract

**Background:**

Compelling evidence claims that gut microbial dysbiosis may be causally associated with major depressive disorder (MDD), with a particular focus on *Alistipes.* However, little is known about the potential microbiota–gut–brain axis mechanisms by which *Alistipes* exerts its pathogenic effects in MDD.

**Methods:**

We collected data from 16S rDNA amplicon sequencing, untargeted metabolomics, and multimodal brain magnetic resonance imaging from 111 MDD patients and 102 healthy controls. We used multistage linked analyses, including group comparisons, correlation analyses, and mediation analyses, to explore the relationships between the gut microbiome (*Alistipes*), fecal metabolome, brain imaging, and behaviors in MDD.

**Results:**

Gut microbiome analysis demonstrated that MDD patients had a higher abundance of *Alistipes* relative to controls. Partial least squares regression revealed that the increased *Alistipes* was significantly associated with fecal metabolome in MDD, involving a range of metabolites mainly enriched for amino acid, vitamin B, and bile acid metabolism pathways. Correlation analyses showed that the *Alistipes*-related metabolites were associated with a wide array of brain imaging measures involving gray matter morphology, spontaneous brain function, and white matter integrity, among which the brain functional measures were, in turn, associated with affective symptoms (anxiety and anhedonia) and cognition (sustained attention) in MDD. Of more importance, further mediation analyses identified multiple significant mediation pathways where the brain functional measures in the visual cortex mediated the associations of metabolites with behavioral deficits.

**Conclusion:**

Our findings provide a proof of concept that *Alistipes* and its related metabolites play a critical role in the pathophysiology of MDD through the microbiota–gut–brain axis.

## Introduction

Major depressive disorder (MDD) is a complex and common mental disorder that affects ~185 million people worldwide (Marx et al., 2023). It is projected that MDD will be ranked as the first cause of global disease burden by 2030 (Malhi & Mann, 2018). MDD seems to be caused by a combined effect of genetic (Kendall et al., 2021; Yuan et al., 2023), environmental (Li et al., 2022), psychological, and biological factors (Drevets, Wittenberg, Bullmore, & Manji, 2022; Fries, Saldana, Finnstein, & Rein, 2023; Won, Na, & Kim, 2022). More recently, the gut microbiota has been posited to play a pivotal role in accounting for the pathogenesis of MDD (Borkent et al., 2022; Goralczyk-Binkowska, Szmajda-Krygier, & Kozlowska, 2022; Marx et al., 2023). It is generally assumed that the gut microbiota is involved in the regulation of mood and behavior through the microbiota–gut–brain axis (Malhi & Mann, 2018; Marx et al., 2023). Compelling evidence has claimed that gut microbial dysbiosis may be causally associated with MDD by affecting the brain (Sanada et al., 2020; J. Yang et al., 2020; P. Zheng et al., 2016).

*Alistipes*, a member of the phylum Bacteroidetes (Shkoporov et al., 2015), is a genus of bacteria with numerous immunological and biochemical pathways that are associated with mental health and disease (Parker, Wearsch, Veloo, & Rodriguez-Palacios, 2020). Clinical studies have demonstrated a higher abundance of the genus *Alistipes* in MDD patients (Caso et al., 2021; Eicher & Mohajeri, 2022; Naseribafrouei et al., 2014; Yamaoka, Uotsu, & Hoshino, 2022). Moreover, a recent Mendelian randomization analysis indicates that there is a causal relationship between the genus *Alistipes* and the development of MDD (Zhao, Baranova, Cao, & Zhang, 2024). Several possible mechanistic explanations have been proposed, including (1) *Alistipes* is an indole-positive organism such that its increase may lead to a decrease in serotonin that is associated with MDD, and/or (2) *Alistipes* can express glutamate decarboxylase that metabolizes glutamate into γ-aminobutyric acid (GABA) such that the increase in *Alistipes* could be related to the increase in GABA. Despite this growing literature, notable gaps remain regarding the potential biological pathways by which *Alistipes* exerts its pathogenic effects in the context of MDD.

The microbiota–gut–brain axis is a complex bidirectional communication system (Cryan & Dinan, 2012; Liu et al., 2023). There are multiple key mediators involved in the communication, such as gut microbiota-derived metabolites. Alterations in the composition of the gut microbiota in MDD might lead to changes in microbial metabolites, which may contribute to the pathophysiology of MDD. For instance, short-chain fatty acids (e.g. acetate, butyrate, and propionate) have been reported to be depleted in MDD patients (Skonieczna-Zydecka et al., 2018), while their administration yields antidepressant effects (Caspani, Kennedy, Foster, & Swann, 2019; van de Wouw et al., 2018). Neurotransmitters (e.g. serotonin and GABA), produced by the gut microbiota directly or indirectly, can influence emotional behavior (Caspani et al., 2019). Levels of the secondary bile acids modulated by the gut microbiota have been shown to be negatively correlated with the severity of depressive symptoms (Sun et al., 2022).

Gut microbiome profiling offers a powerful tool to measure microbial diversity and composition (Claesson, Clooney, & O’Toole, 2017). Metabolomics refers to the high-throughput identification and quantification of small molecular metabolites in biological samples (Clish, 2015; Griffin, Atherton, Shockcor, & Atzori, 2011; Trivedi, Hollywood, & Goodacre, 2017). Since research has identified fecal metabolome as a functional readout of the gut microbiome (Zierer et al., 2018), it is appealing to combine them to reveal changes in both gut microbial composition and function in MDD patients (Liu et al., 2023). In parallel, advancements in brain imaging technologies, particularly multimodal magnetic resonance imaging (MRI), have allowed for the *in vivo* examination of brain structural and functional abnormalities in MDD (Chen et al., 2016, 2022; Fang et al., 2023; Jiang et al., 2017; Sun et al., 2023; Zhao et al., 2021; Zhu et al., 2024). Structural, functional, and diffusion MRI can be leveraged to assess gray matter morphology (Ashburner & Friston, 2000; Dahnke, Yotter, & Gaser, 2013), spontaneous brain function (Tomasi & Volkow, 2010; Zang et al., 2004; Zou et al., 2008), and white matter integrity (Smith et al., 2006). From a methodological point of view, analyzing these multimodal brain imaging measures may provide different yet complementary information on disease neuropathology (Zhu et al., 2022). Collectively, a combined analysis of the above multi-omics data could yield an integrative framework to facilitate a more thorough characterization of the microbiota–gut–brain axis mechanisms underlying MDD (Song et al., 2024).

**Figure 1.**
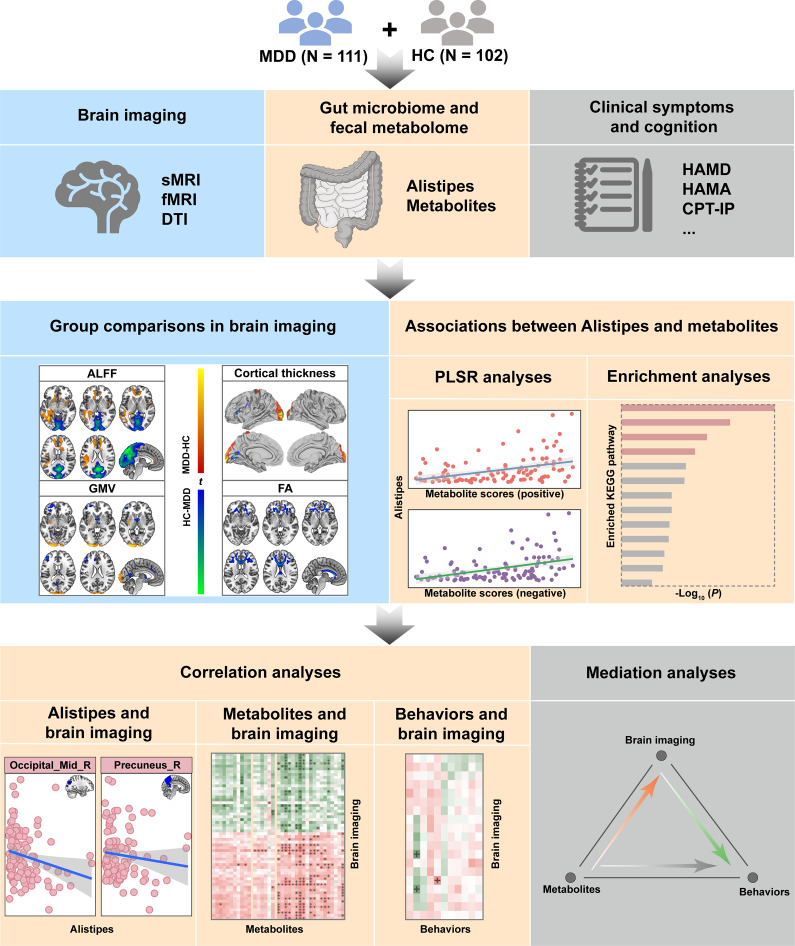
Research design and analytical procedure. We collected data from 16S rDNA amplicon sequencing, untargeted metabolomics, and multimodal brain MRI from 111 MDD patients and 102 HCs. We used multistage linked analyses, including group comparisons, correlation analyses, and mediation analyses, to explore the relationships between the gut microbiome (*Alistipes*), fecal metabolome, brain imaging, and behaviors in MDD. Abbreviations: CPT-IP, Continuous Performance Task-Identical Pairs; DTI, diffusion tensor imaging; fMRI, functional magnetic resonance imaging; HAMA, Hamilton Rating Scale for Anxiety; HAMD, Hamilton Rating Scale for Depression; HCs, healthy controls; MDD, major depressive disorder; PLSR, partial least squares regression; sMRI, structural magnetic resonance imaging.

In this study, we collected data from 16S rDNA amplicon sequencing, untargeted metabolomics, and multimodal brain MRI from 111 MDD patients and 102 healthy controls (HCs). We used multistage linked analyses, including group comparisons, correlation analyses, and mediation analyses, to explore the relationships between the gut microbiome (*Alistipes*), fecal metabolome, brain imaging, and behaviors in MDD. A schematic representation of the research design and analytical procedure is provided in Figure 1.

## Methods

### Participants

The current study enrolled 213 right-handed participants, including 111 MDD patients from the Affiliated Psychological Hospital of Anhui Medical University and 102 HCs from the local community. The diagnoses of MDD were confirmed by two experienced psychiatrists using the MINI-International Neuropsychiatric Interview according to the International Classification of Diseases (ICD-10) criteria. HCs were screened by a detailed interview to ensure an absence of any psychiatric illness. The exclusion criteria for all participants consisted of (1) the presence of other psychiatric disorders such as schizophrenia, bipolar disorder, substance-induced mood disorder, anxiety disorders, substance abuse, or dependence, (2) a history of significant neurological or physical illnesses, (3) a history of head injury with loss of consciousness, (4) any MRI contraindications, including metallic implants and claustrophobia, and (5) taking antibiotics within a month. Additionally, HCs who had a family history of major psychiatric or neurological illnesses among their first-degree relatives were excluded. The 24-item Hamilton Rating Scale for Depression (HAMD) (Hamilton, 1960), the 14-item Hamilton Rating Scale for Anxiety (HAMA), (Hamilton, 1959), and the Beck Depression Inventory (BDI) (Wang & Gorenstein, 2013) were applied to assess the severity of depression and anxiety symptoms. The Revised Social Anhedonia Scale (RSAS) (Hu et al., 2018), the Revised Physical Anhedonia Scale (RPAS) (Kollias et al., 2008), and the Temporal Experience of Pleasure Scale (TEPS) (Zhou et al., 2019) were used to assess the severity of anhedonia. With respect to cognition, the Continuous Performance Task-Identical Pairs (CPT-IP) task was used to measure sustained attention (Cornblatt et al., 1988), with CPT-IP-2, CPT-IP-3, and CPT-IP-4 corresponding to different loads, and the Digital Span test (forward and backward) (Groth-Marnat & Baker, 2003) was used to measure working memory. All patients were receiving their regular antidepressant medications, including selective serotonin reuptake inhibitors, serotonin-norepinephrine reuptake inhibitors, or noradrenergic and specific serotonergic antidepressants. This study was approved by the ethics committee of the First Affiliated Hospital of Anhui Medical University. Written informed consent was obtained from all participants after they had been given a complete description of the study.

### Image acquisition

MRI data were acquired on two 3.0-Tesla MRI scanners (Discovery MR750w and MR750, General Electric, Milwaukee, WI, USA) using the same protocol. During scanning, participants were instructed to relax, keep their eyes closed but not fall asleep, think of nothing in particular, and move as little as possible. All participants underwent a high-resolution three-dimensional T1-weighted brain volume (BRAVO) sequence with the following parameters: repetition time (TR) = 8.5 ms; echo time (TE) = 3.2 ms; inversion time (TI) = 450 ms; flip angle = 12°; field of view (FOV) = 256 mm × 256 mm; matrix size = 256 × 256; slice thickness = 1 mm, no gap; voxel size = 1 mm × 1 mm × 1 mm; 188 sagittal slices. Resting-state blood-oxygen-level-dependent (BOLD) functional MRI (fMRI) data were acquired using a gradient-echo single-shot echo planar imaging sequence with the following parameters: TR = 2,000 ms; TE = 30 ms; FA = 90°; FOV = 220 mm × 220 mm; matrix size = 64 × 64; slice thickness = 3 mm, slice gap = 1 mm; 185 volumes. Diffusion tensor imaging (DTI) data were acquired by a spin-echo single-shot echo planar imaging sequence with the following parameters: TR = 10,000 ms; TE = 74 ms; FA = 90°; FOV = 256 mm × 256 mm; matrix =128 × 128; slice thickness = 3 mm without gap; 50 axial slices; 64 diffusion gradient directions (*b* = 1,000 s/mm^2^) plus five *b* = 0 reference images. Routine T2-weighted images were also collected to exclude any organic brain abnormality. None of the participants were excluded for visually inspected imaging artifacts.

### Structural MRI data analysis

Gray matter morphology was assessed using voxel-based morphometry (VBM) and surface-based morphometry (SBM) analyses. VBM analysis was performed using the CAT12 toolbox (http://www.neuro.uni-jena.de/cat) implemented in Statistical Parametric Mapping (SPM12; http://www.fil.ion.ucl.ac.uk/spm). First, all the structural T1-weighted images were corrected for bias-field inhomogeneities. Second, these images were segmented into gray matter, white matter, and cerebrospinal fluid density maps using the ‘new-segment’ approach (Ashburner & Friston, 2005), with total intracranial volume (TIV) obtained. Third, a diffeomorphic anatomical registration through the exponentiated Lie algebra (DARTEL) technique was used to generate a custom, study-specific template (Ashburner, 2007). Fourth, each participant’s gray matter density image was warped to the customized template; then the resultant images were affine registered to the Montreal Neurological Institute (MNI) space and resampled to a voxel size of 1.5 mm × 1.5 mm × 1.5 mm. Fifth, modulation was applied by multiplying the transformed gray matter density maps with the nonlinear components of Jacobian determinants, resulting in the normalized gray matter volume (GMV) maps. Finally, the GMV maps were smoothed with a 6-mm full-width at half-maximum (FWHM) Gaussian kernel.

SBM analysis was performed using the surface-processing pipeline of the CAT12 toolbox. Two commonly used measures – cortical thickness (CT) and sulcus depth (SD) – were calculated. Specifically, a projection-based thickness estimation approach was used for the reconstruction of the central surface and the calculation of CT (Dahnke et al., 2013). For the estimation of white matter distances, we subjected the T1-weighted images to tissue segmentation. Local maxima were then projected to other gray matter voxels by using a neighbor relationship described by the white matter distance. These values equal CT. This projection-based approach also included partial volume correction and correction for sulcal blurring and asymmetries. Topological correction was carried out through a method based on spherical harmonics. For the reparametrization of the surfaces, an algorithm for spherical mapping of the cortical surface was applied (Yotter, Dahnke, Thompson, & Gaser, 2011). An adapted two-dimensional DARTEL algorithm (Ashburner, 2007) was then applied to the surface for spherical registration. SD was extracted based on the Euclidean distance between the central surface and its convex hull and then transformed with the sqrt function (Lohmann, 1998; Yotter et al., 2011). Finally, 15-mm and 20-mm FWHM Gaussian kernels were used to smooth the resampled CT and SD maps, respectively.

### Resting-state fMRI data analysis

Resting-state fMRI data were preprocessed using the Data Processing & Analysis for Brain Imaging (http://rfmri.org/dpabi) software (Yan, Wang, Zuo, & Zang, 2016) according to a validated pipeline (Cheng et al., 2025; Mo et al., 2024; Zhang et al., 2024). The first 10 volumes for each participant were removed to allow the signal to reach equilibrium and the participants to adapt to the scanning noise. The remaining volumes were corrected for the acquisition time delay between slices. Then, realignment was performed to correct the motion between time points. Head motion parameters were computed by estimating the translation in each direction and the angular rotation on each axis for each volume. All BOLD data were within the defined motion thresholds (i.e. translational or rotational motion parameters less than 2.5 mm or 2.5°). We also calculated frame-wise displacement (FD), which indexes the volume-to-volume changes in head position. Several nuisance covariates (the linear drift, the estimated motion parameters based on the Friston-24 model, the spike volumes with FD >0.5 mm, the white matter signal, and the cerebrospinal fluid signal) were regressed out of the data. Notably, we did not perform global signal regression since it is still a controversial topic in resting-state fMRI analysis (Murphy & Fox, 2017). The datasets were then band-pass filtered on a frequency range of 0.01–0.1 Hz. For spatial normalization, individual structural images were first co-registered with the mean functional images; then the transformed structural images were segmented and normalized to the MNI space using the DARTEL technique (Ashburner, 2007). Finally, each filtered functional volume was spatially normalized to the MNI space using the deformation parameters estimated during the above step and resampled into a 3-mm cubic voxel.

Resting-state fMRI measures reflecting spontaneous brain function were calculated in the following way (Zhu et al., 2024): (i) The calculation of the amplitude of low-frequency fluctuations (ALFF) and the fractional ALFF (fALFF) was based on the preprocessed fMRI data without band-pass filtering. Specifically, the BOLD time course of each voxel was transformed to a frequency domain via a Fast Fourier Transform to obtain the power spectrum. The ALFF was defined as the average square root in a specific low-frequency band (0.01–0.1 Hz) (Zang et al., 2007). The fALFF was defined as the ratio of the power spectrum in the low-frequency band (0.01–0.1 Hz) to that in the entire frequency range (Zou et al., 2008). (ii) The regional homogeneity (ReHo) was calculated as Kendall’s concordance coefficient of the time course of a given voxel with those of its nearest neighbors (26 voxels) (Yang et al., 2020; Zang et al., 2004). (iii) For a given voxel, the functional connectivity density (FCD) was defined as the number of functional connections with correlation coefficients above a threshold of 0.25 between that voxel and all other voxels within the whole brain (Cui et al., 2023; Zhu et al., 2017). (iv) The voxel-mirrored homotopic connectivity (VMHC) was defined as the functional connectivity between any pair of symmetric inter-hemispheric voxels, that is, Pearson’s correlation coefficient between the time course of each voxel and that of its symmetrical inter-hemispheric counterpart (S. Liu et al., 2024; Zuo et al., 2010). For all the above fMRI measures, the value of each voxel was rescaled to a whole-brain mean of 1, yielding the standardized maps that were spatially smoothed with a 6-mm FWHM Gaussian kernel.

### DTI data analysis

For DTI data, standard processing steps were performed by using the FMRIB Software Library (FSL; www.fmrib.ox.ac.uk/fsl). First, eddy current distortion and head motion were corrected by registering the diffusion-weighted images to the first *b*0 image through the affine transformations. Second, the data were skull-stripped by using the FMRIB Brain Extraction Tool. By using the DTIFIT toolbox, diffusion parameters, including fractional anisotropy (FA), mean diffusivity (MD), axial diffusivity (AD), and radial diffusivity (RD), were calculated to evaluate white matter integrity. Then, the tract-based spatial statistics (TBSS) pipeline was conducted (Smith et al., 2006). Briefly, individual FA images were first nonlinearly registered to the MNI space. After transformation into the MNI space, a mean FA image was created and thinned to generate a mean FA skeleton. Then, each subject’s FA image was projected onto the skeleton by filling the mean FA skeleton with FA values from the nearest relevant tract center by searching perpendicular to the local skeleton structure for maximum FA value. Finally, the registration and projection information derived from the FA analysis was applied to the other diffusion parameters to project MD, AD, and RD images onto this common skeleton.

### Gut microbiome analysis

Fecal samples were collected and stored within 1 day before or after the MRI examination. The fecal sample was aliquoted using a scoop into a sterilized tube and stored at −80 °C immediately after collection. The gut microbiome was quantified using 16S rRNA gene amplicon sequencing. Briefly, total genome DNA from the fecal samples was extracted using the cetyltrimethylammonium bromide method. To construct the polymerase chain reaction (PCR)-based 16S rDNA amplicon library for sequencing, PCR enrichment of the V4 hypervariable region of 16S rDNA was performed with the forward primer 515F (5′-GTGCCAGCMGCCGCGGTAA-3′) and reverse primer 806R (5′-GGACTACHVGGGTWTCTAAT-3′). Sequencing libraries were generated using the TruSeq^®^ DNA PCR-Free Sample Preparation Kit (Illumina, USA) following the manufacturer’s recommendations, and index codes were added. The library quality was assessed on the Qubit@2.0 Fluorometer (Thermo Scientific) and the Agilent Bioanalyzer 2100 system. At last, the library was sequenced on an Illumina NovaSeq platform, and 250-bp paired-end reads were generated.

We used a standard pipeline for 16S rDNA data analysis (Liu et al., 2021; Song et al., 2024). First, the raw paired-end reads were assigned to samples based on their unique barcode sequences. Then, the paired-end reads were merged to obtain raw tags based on the 3′ overlapping regions, and the barcode and primers were removed. Quality filtering was performed on the raw tags, retaining reads with error rates below 1%. Next, denoising was done by the unoise3 command, which is an implementation of the UNOISE algorithm. After dereplication, denoised sequences were generated and referred to as amplicon sequence variants (ASVs). Finally, reference-based chimera detection was conducted using the Ribosomal Database Project as a reference database (http://rdp.cme.msu.edu), and then the ASV table was generated by quantifying the normalized ASV counts in each sample. The ASVs were classified at different taxonomical units from the genus to the phylum level, with a minimum confidence threshold of 0.1. After removing plastid and non-bacteria, microbial relative abundance at the genus level was obtained.

### Fecal metabolomics analysis

The fecal metabolome was analyzed using ultrahigh-performance liquid chromatography coupled with a tandem mass spectrometry system (Barri & Dragsted, 2013; Want et al., 2006). A pooled quality control (QC) sample and instrument blanks were used to assess the reproducibility and to filter out the chemical background contamination. The raw data were processed by MS-DIAL (version 4.92) (Tsugawa et al., 2015), with a mass tolerance of 0.01 and 0.025 Da for MS1 and MS2, respectively; the minimum peak height was 50,000, and the identification score cut-off was 70%. For the metabolite identification process, we used the spectral libraries provided by the MS-DIAL team, which incorporate all the available public repositories. The average QC to blank peak area ratio was calculated, and metabolites with a QC/blank peak area ratio <3 were removed (Gadara et al., 2021). Signal reproducibility was tested by calculating the relative standard deviation (RSD) of QC sample technical replicates, and metabolites with RSD >30% were discarded (Harshfield et al., 2022). Metabolites with nonzero measurements in at least 80% of the samples were included (Bijlsma et al., 2006). Missing values were replaced by one-fifth of the minimum positive values of their corresponding variables. Finally, the quantification values of metabolites were normalized by QC samples, made more normally distributed with a generalized log transformation, and standardized using *z*-scores.

### Statistical analysis

Demographic, clinical, and cognitive data between MDD patients and HCs were analyzed with the SPSS 25.0 software (SPSS, Chicago, IL, USA) using a two-sample *t*-test or chi-square test, as appropriate. A threshold of *P* < 0.05 was considered statistically significant (two-sided).

We tested differences in brain imaging measures between MDD patients and HCs. Voxel-based group comparisons in GMV, ALFF, fALFF, ReHo, FCD, and VMHC were conducted using two-sample *t*-tests in the SPM12 software. Vertex-based group comparisons in CT and SD were carried out using two-sample *t*-tests in the CAT12 toolbox. Multiple comparisons were corrected using a cluster-level family-wise error (FWE) method, resulting in a cluster-defining threshold of *P* = 0.001 and a corrected cluster significance of *P* < 0.05. For TBSS-based group comparisons in the four DTI parameters (FA, MD, AD, and RD), the nonparametric permutation testing (permutation number = 5,000) and threshold-free cluster enhancement in the FSL software were used for statistical inference. The FWE method was also used to correct for multiple comparisons. If a brain imaging measure exhibited a significant group difference in a cluster, the mean value within this cluster was extracted for subsequent correlation and mediation analyses.

To establish the relationships between gut microbiota, fecal metabolites, brain imaging, and behaviors, we adopted a multistage approach. First, the Mann–Whitney *U* test was used to compare the relative abundance of *Alistipes* between MDD patients and HCs. Second, partial least squares regression (PLSR) was utilized to investigate the associations between *Alistipes* and metabolites in MDD patients. PLSR identified the first component where the weighted sum of metabolites (i.e. metabolite scores) had the maximum covariance with *Alistipes.* Pearson’s correlation coefficient between *Alistipes* and the metabolite scores was calculated to reflect the overall *Alistipes*–metabolites association. The statistical significance of the association was evaluated with nonparametric permutation testing by randomly shuffling the participants 5,000 times. Furthermore, the contribution of an individual metabolite was assessed by its contribution loading, that is, Pearson’s correlation coefficient between this metabolite and the PLSR metabolite scores. Strongly contributing metabolites were defined as those among the top 20% of metabolites with positive and negative loadings, which were termed PLSR+ and PLSR− metabolites, respectively. Then, we performed enrichment analysis for the PLSR+ and PLSR− metabolites based on the Kyoto Encyclopaedia of Genes and Genomes database (https://www.kegg.jp/kegg/pathway.html). Third, we examined the associations of the differential brain imaging measures with *Alistipes*, the PLSR+ and PLSR− metabolites, and behaviors (clinical symptoms and cognition) using partial Spearman’s correlation analyses adjusting for age, gender, education, and site distribution (for GMV, we additionally adjusted for TIV; for ALFF, fALFF, ReHo, FCD, and VMHC, we additionally adjusted for FD). For the abovementioned correlation analyses, multiple testing was corrected using the false discovery rate (FDR) method, with a corrected significance level of *P* < 0.05. Finally, based on the correlation results, we further performed a mediation analysis to test whether brain imaging (M) mediated the associations between metabolites (X) and behaviors (Y) in MDD patients. In the mediation model, the total effect of X on Y (*c*) = indirect effect of X on Y through M (*a* × *b*) + direct effect of X on Y (*c′*). The significance analysis was based on 5,000 bootstrap realizations, and a significant indirect effect is indicated when the bootstrap 95% confidence interval (CI) does not include zero. Age, gender, education, site distribution, and TIV/FD were considered nuisance variables.

### Validation analysis

To test the possible effects of antidepressant medication, illness duration, and body mass index (BMI) on our results, we included these factors as additional nuisance covariates in the correlation and mediation analyses.

## Results

### Demographic, clinical, and cognitive characteristics

Demographic, clinical, and cognitive characteristics of the sample are provided in Table 1. There were no significant group differences in age, gender, site distribution, and BMI, but MDD patients had a lower educational level than HCs. Regarding clinical and cognitive variables, MDD patients had higher scores on the HAMD, HAMA, BDI, RSAS, and RPAS, and lower scores on the TEPS, CPT-IP-2, CPT-IP-3, CPT-IP-4, DST-forward, and DST-backward than HCs.

**Table 1.** Demographic, clinical, and cognitive characteristics of the participants

Characteristics	MDD	HCs	Statistics	*P*-value
Number of subjects	111	102		
Gender (female/male)	67/44	61/41	*χ*^2^ = 0.007	0.934
Site distribution	67/44	61/41	*χ*^2^ = 0.007	0.934
Age (years)	36.86 ± 13.38	34.70 ± 11.17	*t* = 1.288	0.199
Education (years)	12.35 ± 3.61	15.25 ± 4.66	*t* = −5.052	<0.001
BMI (kg/m^2^)	22.57 ± 3.81	22.84 ± 3.11	*t* = −0.558	0.578
Illness duration (years)	4.76 ± 6.89			
TIV (mm^3^)	1,460.10 ± 121.63	1,472.58 ± 129.46	*t* = −0.725	0.469
FD (mm)	0.11 ± 0.05	0.13 ± 0.06	*t* = −2.808	0.005
HAMD	31.97 ± 8.67	2.10 ± 3.24	*t* = 32.431	<0.001
HAMA	19.15 ± 6.89	0.87 ± 2.28	*t* = 25.532	<0.001
BDI	27.64 ± 10.91	4.04 ± 4.20	*t* = 21.157	<0.001
RSAS	21.32 ± 6.90	12.57 ± 6.33	*t* = 8.185	<0.001
RPAS	27.46 ± 9.38	17.98 ± 12.08	*t* = 5.310	<0.001
TEPS	60.38 ± 16.37	74.34 ± 12.69	*t* = −6.213	<0.001
CPT-IP–2	2.49 ± 0.97	2.95 ± 0.99	*t =* −2.920	0.004
CPT-IP–3	1.76 ± 0.88	2.33 ± 1.05	*t =* −3.806	<0.001
CPT-IP–4	1.07 ± 0.67	1.58 ± 0.84	*t =* −4.383	<0.001
DST-forward	8.18 ± 1.47	8.76 ± 1.20	*t =* −3.172	0.002
DST-backward	5.08 ± 1.69	5.93 ± 1.52	*t =* −3.844	<0.001

*Note:* Except for gender and site distribution, data are expressed as mean ± standard deviation.

Abbreviations: BDI, Beck Depression Inventory; BMI, body mass index; CPT-IP, Continuous Performance Task-Identical Pairs; DST, Digital Span test; FD, frame-wise displacement; HAMA, Hamilton Rating Scale for Anxiety; HAMD, Hamilton Rating Scale for Depression; HCs, healthy controls; MDD, major depressive disorder; RPAS, Revised Physical Anhedonia Scale; RSAS, Revised Social Anhedonia Scale; TEPS, Temporal Experience of Pleasure Scale; TIV, total intracranial volume.

### Brain structural and functional abnormalities in MDD

**Figure 2.**
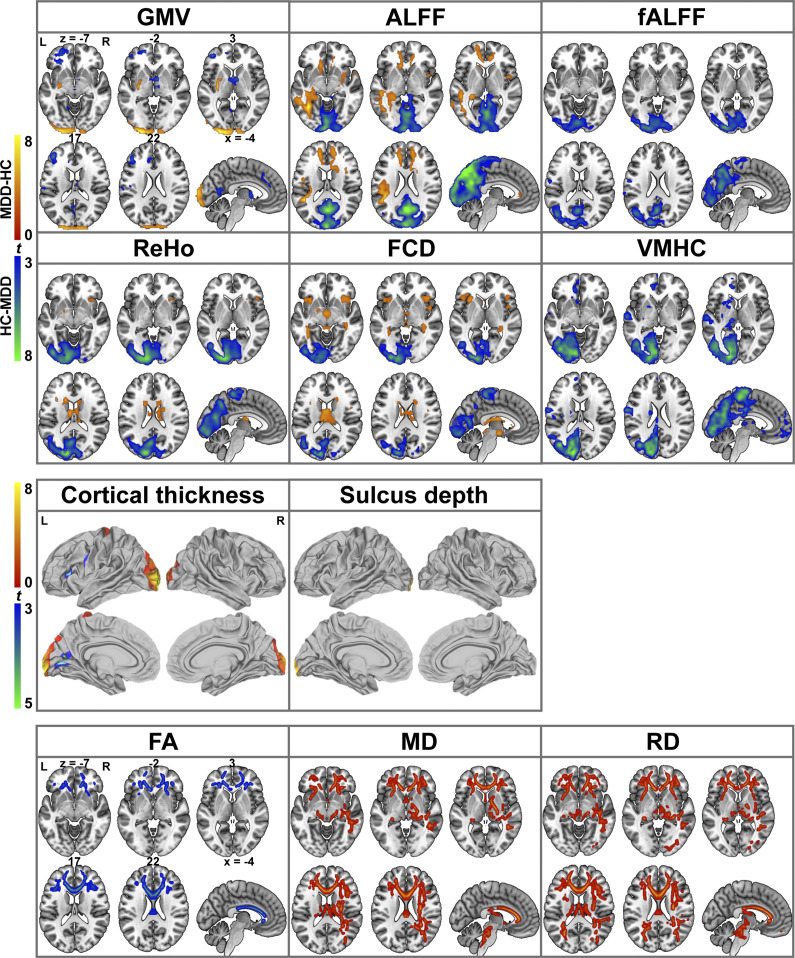
Brain imaging differences between MDD patients and HCs. Abbreviations: ALFF, amplitude of low-frequency fluctuations; FA, fractional anisotropy; fALFF, fractional amplitude of low-frequency fluctuations; FCD, functional connectivity density; GMV, gray matter volume; HCs, healthy controls; L, left; MD, mean diffusivity; MDD, major depressive disorder; R, right; RD, radial diffusivity; ReHo, regional homogeneity; VMHC, voxel-mirrored homotopic connectivity.

Significant differences in brain imaging measures between MDD patients and HCs are illustrated in Figure 2 (*P* < 0.05, FWE-corrected). Overall, there were widespread brain structural and functional abnormalities in MDD. With respect to gray matter morphology, MDD patients exhibited a mixture of decreased and increased GMV/CT, as well as increased SD. In regard to spontaneous brain function, MDD patients presented a mix of decreased and increased ALFF/ReHo/FCD, as well as decreased fALFF/VMHC. Regarding white matter integrity, MDD patients showed decreased FA and increased MD/RD.

### Associations between Alistipes and fecal metabolites in MDD

**Figure 3.**
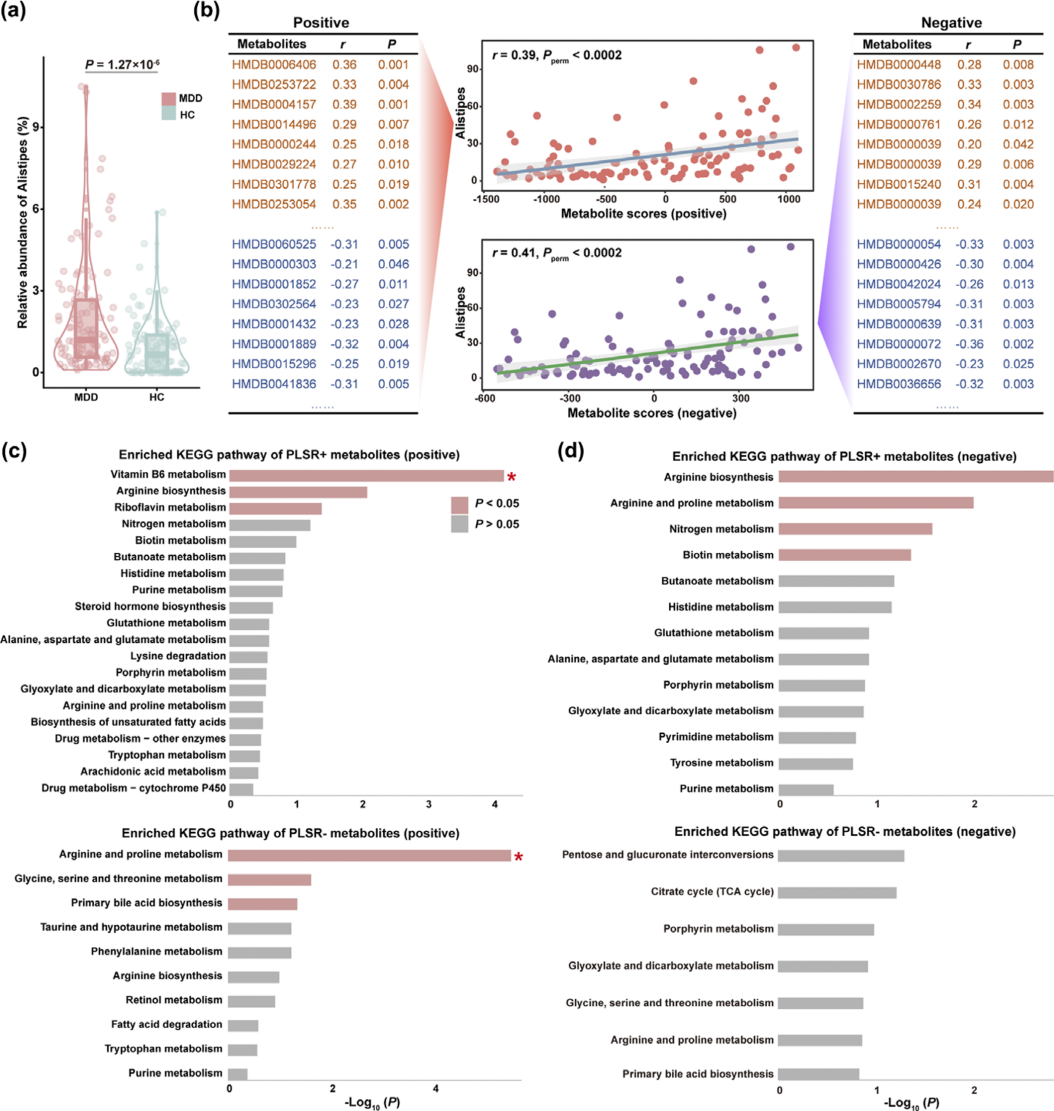
Associations between *Alistipes* and fecal metabolites in MDD. (a) MDD patients had a higher abundance of *Alistipes* compared with HCs. (b) Under both the positive and negative ion collection modes, PLSR analysis identified significant overall correlations between *Alistipes* and metabolites in MDD patients. Metabolites were ranked by their contribution loadings, and strongly contributing metabolites were defined as those among the top 20% of metabolites with positive and negative loadings (orange PLSR+ and blue PLSR− metabolites). (c,d) Further enrichment analysis of the PLSR+ and PLSR− metabolites under the positive and negative ion collection modes. The *y*-axis represents the KEGG metabolic pathway, and the *x*-axis denotes −log_10_(*P*) with the *P*-value indicating enrichment statistical significance. *P < 0.05, FDR-corrected. Abbreviations: FDR, false discovery rate; HCs, healthy controls; KEGG, Kyoto Encyclopaedia of Genes and Genomes; MDD, major depressive disorder; PLSR, partial least squares regression.

In line with our expectation, MDD patients had a higher abundance of *Alistipes* compared with HCs (*z* = 4.845, *P* = 1.27 × 10^−6^) (Figure 3a). Under both the positive and negative ion collection modes, PLSR analysis identified significant overall correlations between *Alistipes* and fecal metabolites in MDD patients (*r*_pos_ = 0.39, *P*_perm_ < 0.0002; *r*_neg_ = 0.41, *P*_perm_ < 0.0002) (Figure 3b). Metabolites were ranked by their contribution loadings (Supplementary Material), and strongly contributing metabolites were defined as those among the top 20% of metabolites with positive and negative loadings (the PLSR+ and PLSR− metabolites). Further enrichment analysis demonstrated that the PLSR+ and PLSR− metabolites were significantly enriched for amino acid, vitamin B, and bile acid metabolism pathways (Figure 3c,d).

### Associations of brain imaging with Alistipes, metabolites, and behaviors in MDD

**Figure 4.**
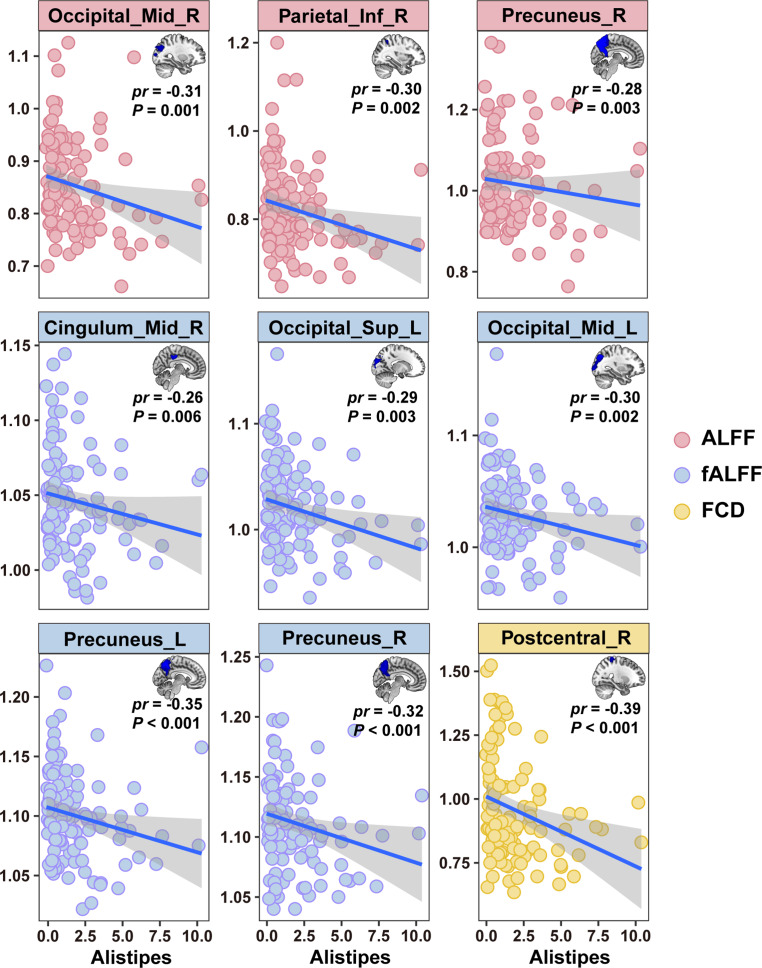
Associations between *Alistipes* and brain imaging in MDD. Abbreviations: ALFF, amplitude of low-frequency fluctuations; fALFF, fractional amplitude of low-frequency fluctuations; FCD, functional connectivity density; Inf, inferior; L, left; MDD, major depressive disorder; Mid, middle; *pr*, partial correlation coefficient; R, right; Sup, superior.

**Figure 5.**
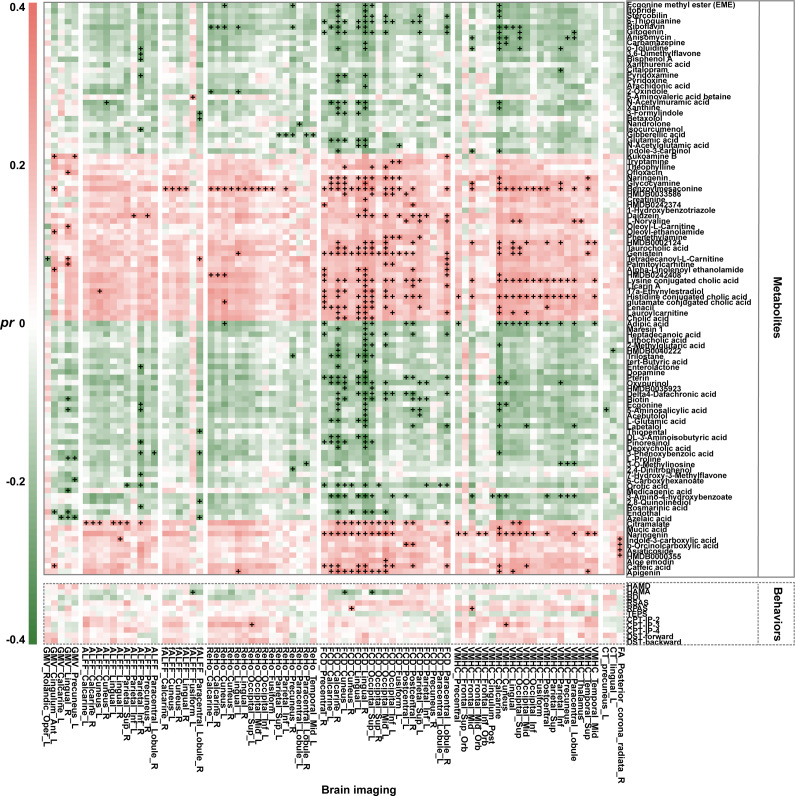
Heatmap showing the associations of brain imaging with metabolites and behaviors in MDD. The color represents the partial correlation coefficient. ^**+**^*P* < 0.05, FDR-corrected. Abbreviations: ALFF, amplitude of low-frequency fluctuations; Ant, anterior; BDI, Beck Depression Inventory; CPT-IP, Continuous Performance Task-Identical Pairs; CT, cortical thickness; DST, Digital Span test; FA, fractional anisotropy; fALFF, fractional amplitude of low-frequency fluctuations; FCD, functional connectivity density; FDR, false discovery rate; GMV, gray matter volume; HAMA, Hamilton Rating Scale for Anxiety; HAMD, Hamilton Rating Scale for Depression; HCs, healthy controls; Inf, inferior; L, left; MD, mean diffusivity; MDD, major depressive disorder; Mid, middle; *pr*, partial correlation coefficient; R, right; ReHo, regional homogeneity; RPAS, Revised Physical Anhedonia Scale; RSAS, Revised Social Anhedonia Scale; TEPS, Temporal Experience of Pleasure Scale; Sup, superior; VMHC, voxel-mirrored homotopic connectivity.

In MDD patients, *Alistipes* was negatively correlated with the differential brain imaging measures, including ALFF of the right middle occipital gyrus, right inferior parietal lobe and right precuneus, fALFF of the right middle cingulate gyrus, left superior occipital gyrus, left middle occipital gyrus and bilateral precuneus, and FCD of the right postcentral gyrus (*P* < 0.05, FDR-corrected) (Figure 4). A wide array of significant correlations were observed between the PLSR+ and PLSR− metabolites and the differential brain imaging measures (*P* < 0.05, FDR-corrected) (Figure 5). For example, cholic acid was positively correlated with FCD of the right calcarine sulcus, left cuneus, bilateral lingual gyrus, and left superior occipital gyrus; carbamazepine was negatively correlated with FCD of the right lingual gyrus and VMHC of the calcarine and cuneus; and benzoylmesaconine was positively correlated with fALFF of the right calcarine sulcus, FCD of the bilateral calcarine sulcus, ReHo of the bilateral lingual gyrus, VMHC of the cuneus, and GMV of the left anterior cingulate gyrus. In addition, we found significant correlations between some behavioral variables and the differential brain imaging measures in MDD patients (*P* < 0.05, FDR-corrected) (Figure 5). That said, HAMA was negatively correlated with fALFF of the left fusiform gyrus and FCD of the left cuneus and left superior occipital gyrus; RPAS was positively correlated with FCD of the right cuneus and VMHC of the middle frontal gyrus; and CPT-IP-3 was positively correlated with ReHo of the left superior occipital gyrus and VMHC of the cuneus.

### Relationships among metabolites, brain imaging, and behaviors in MDD

**Figure 6.**
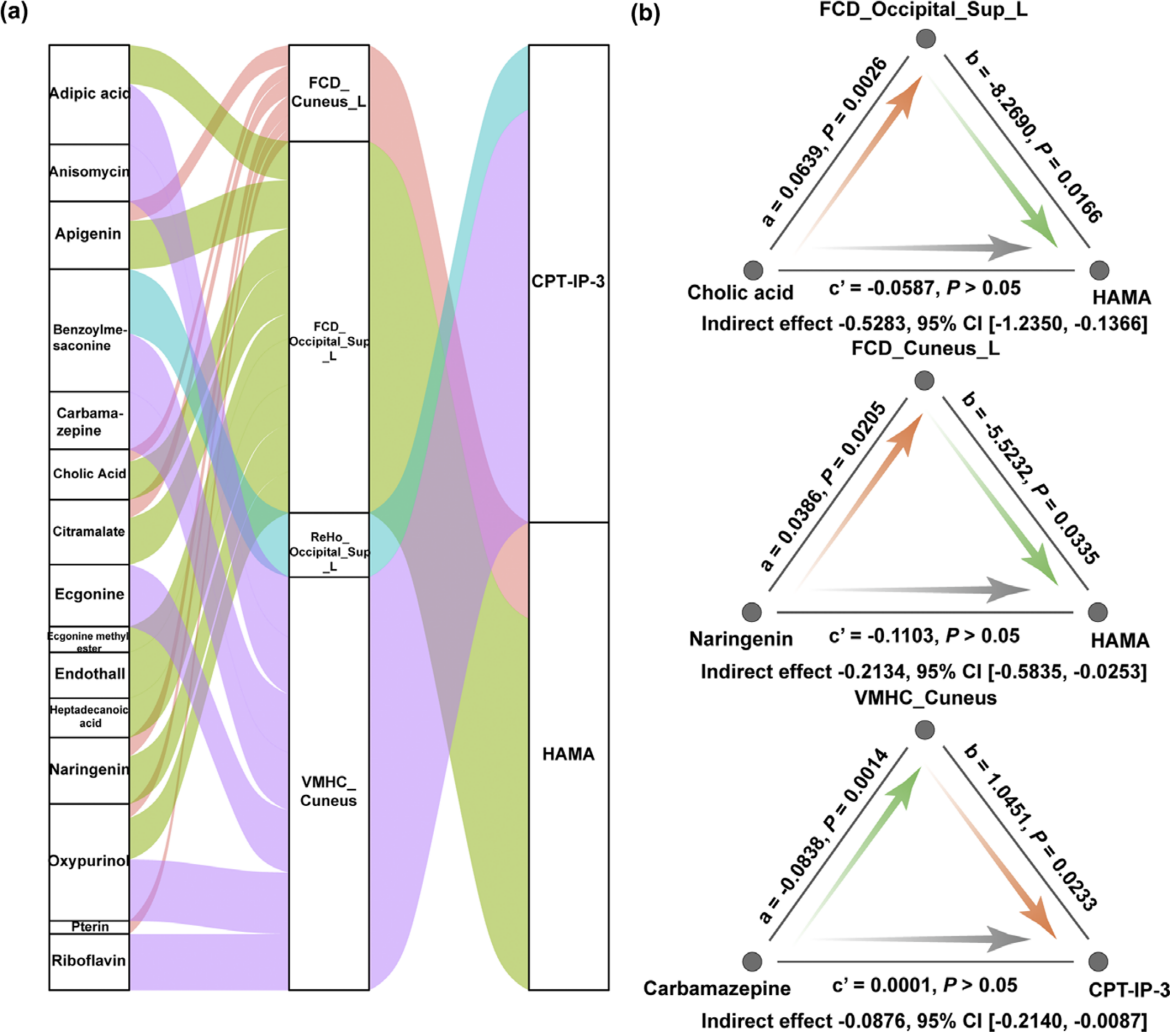
Metabolites–brain imaging–behaviors relationships in MDD. (a) Parallel coordinates chart showing 23 significant mediation pathways where brain functional measures in the visual cortex (middle) mediated the associations of metabolites (left) with affective symptoms and cognition (right) in MDD patients. The curved lines connecting the panels indicate the mediation effects, with colors corresponding to different brain functional measures. (b) Graphical representation of three representative mediation pathways. In the mediation model, the total effect of metabolites on behaviors (*c*) = indirect effect through brain imaging (*a* × *b*) + direct effect (*c′*). Abbreviations: CI, confidence interval; CPT-IP, Continuous Performance Task-Identical Pairs; FCD, functional connectivity density; HAMA, Hamilton Rating Scale for Anxiety; L, left; MDD, major depressive disorder; ReHo, regional homogeneity; Sup, superior; VMHC, voxel-mirrored homotopic connectivity.

Based on the abovementioned correlation results, we further carried out a mediation analysis to test whether brain imaging mediated the associations between metabolites and behaviors. As shown in Figure 6a, we found 23 significant mediation pathways where brain functional measures in the visual cortex mediated the associations of metabolites with affective symptoms and cognition in MDD patients. For instance, the association between cholic acid and HAMA was significantly mediated by FCD of the left superior occipital gyrus (indirect effect = −0.5283, 95% CI = −1.2350 to −0.1366); the association between naringenin and HAMA was significantly mediated by FCD of the left cuneus (indirect effect = −0.2134, 95% CI = −0.5835 to −0.0253); and the association between carbamazepine and CPT-IP-3 was significantly mediated by VMHC of the cuneus (indirect effect = −0.0876, 95% CI = −0.2140 to −0.0087) (Figure 6b).

### Validation analysis

After additionally adjusting for antidepressant types, illness duration, and BMI, the associations of brain imaging with *Alistipes*, metabolites, and behaviors in MDD patients were largely preserved (Table S1 and Figure S1 in the Supplementary Material). Moreover, the results of the metabolites–brain imaging–behaviors mediation analysis partially held after additionally controlling for these factors (Figure S2 in the Supplementary Material).

## Discussion

The present study provides preliminary evidence that *Alistipes* and its related metabolites play a critical role in the pathophysiology of MDD through the microbiota–gut–brain axis. First, gut microbiome analysis demonstrated that MDD patients had a higher abundance of *Alistipes* relative to HCs. PLSR revealed that the increased *Alistipes* was significantly associated with fecal metabolome in MDD, involving a range of metabolites mainly enriched for amino acid, vitamin B, and bile acid metabolism pathways. Correlation analyses showed that the *Alistipes*-related metabolites were associated with a wide array of brain imaging measures involving gray matter morphology, spontaneous brain function, and white matter integrity, among which the brain functional measures were, in turn, associated with affective symptoms (anxiety and anhedonia) and cognition (sustained attention) in MDD. Of more importance, further mediation analyses identified multiple significant mediation pathways where the brain functional measures in the visual cortex mediated the associations of metabolites with behavioral deficits.

Consistent with previous research, we found that the abundance of *Alistipes* was elevated in MDD patients compared to HCs (Jiang et al., 2015; Naseribafrouei et al., 2014). The genus *Alistipes*, a member of the phylum Bacteroidetes, is Gram-negative, strictly anaerobic, and indole-positive (Song et al., 2006). *Alistipes* could yield reactions with tryptophan and may thus influence tryptophan availability (Muller, 1986). As tryptophan is the precursor to serotonin, an increased abundance of *Alistipes* in MDD might disrupt the balance in the serotonergic system. It is widely recognized that the dysregulation of serotonin is a major contributing factor in the development of MDD, and many antidepressants exert their effects by targeting the serotonin system (Cardon et al., 2024). Given the correlation between elevated abundance of *Alistipes* and an increased incidence of abdominal pain in patients with IBS, a hypothesis has been proposed that *Alistipes* may be linked to gut inflammation (Saulnier et al., 2011). Nevertheless, further investigation is warranted to elucidate the role of *Alistipes* in inflammation and depression. In addition, animal research has shown that *Alistipes* can express glutamate decarboxylase, an enzyme that metabolizes glutamate into GABA (Polansky et al., 2015). This invites the speculation that increased *Alistipes* may lead to increased GABA in MDD patients, as evidence has indicated the importance of GABAergic inhibitory neurotransmission in the neuropathology of depression (Luscher, Maguire, Rudolph, & Sibille, 2023).

In MDD patients, increased *Alistipes* abundance was found to be associated with a range of fecal metabolites, mainly enriched in amino acid, vitamin B, and bile acid metabolism pathways. The intimate link between abnormal amino acid metabolism and MDD has been well established. For instance, Ho, Tay, Wee, & Ching (2023) reported elevated serum levels of several amino acid metabolites in MDD patients compared with HCs, including glutamic acid, aspartic acid, and glycine, among which glutamic acid levels were further correlated with depression severity. Moreover, these metabolites could act as potential diagnostic biomarkers as they could better distinguish between depressed and healthy individuals (Ho et al., 2023). Remarkably, glutamate is the primary excitatory neurotransmitter in the brain, and strong evidence implicates the glutamate system in pathophysiological processes that are relevant to MDD (Murrough, Abdallah, & Mathew, 2017). Glutamate levels have been shown to be elevated in the plasma, cerebrospinal fluid, and the brains of MDD patients (Sanacora, Zarate, Krystal, & Manji, 2008). Genetic research has also supported an association between glutamate-related gene variants and MDD (Smoller, 2016). Vitamins are necessary for normal physical function and mental health. The B vitamins serve as pivotal micronutrients to maintain brain function based on their general metabolic functions and their roles in neurochemical synthesis (Kennedy, 2016). Riboflavin metabolism abnormality may cause dysregulation of several cellular enzymatic processes, which would have broad negative consequences for brain function (Kennedy, 2016). Moreover, empirical evidence has emphasized the important roles of folates and vitamin B12 in MDD and its intervention (Borges-Vieira & Cardoso, 2023; Reynolds, 2006; W. Zheng et al., 2020). Cholic acid, physiologically a predominant primary bile acid, is the main product of cholesterol degradation in the liver and is metabolized in the intestine by the gut microbiota (Wahlstrom, Sayin, Marschall, & Backhed, 2016). It can promote the absorption and transport of lipids in the gut, maintain cholesterol homeostasis, and influence energy expenditure (Vaz & Ferdinandusse, 2017), which may be directly or indirectly related to the pathophysiology of depression. An animal metabonomics study found that depressed rats had markedly elevated levels of cholic acid in plasma (F. Zhang et al., 2010). Clinical research has demonstrated that MDD patients may have significant differences in cholesterol levels relative to healthy controls and has further pointed out the adverse impact of hypercholesterolemia on the treatment of MDD (Papakostas et al., 2004). Moreover, a review highlights that abnormal lipid metabolism in the brain may influence the onset and progression of MDD by affecting neurogenesis and synaptic formation (Du et al., 2016).

Our multimodal MRI data showed that the *Alistipes*-related metabolites were associated with a wide array of brain imaging measures involving gray matter morphology, spontaneous brain function, and white matter integrity, among which the brain functional measures were in turn associated with affective symptoms (anxiety and anhedonia) and cognition (sustained attention) in MDD. Of more importance, further mediation analyses identified multiple significant mediation pathways where the brain functional measures in the visual cortex mediated the associations of metabolites with behavioral deficits. On the one side, these findings imply that *Alistipes* and its related metabolites may influence diverse properties of brain structure and function that are broadly distributed across distinct brain systems. On the other side, the current observation of the mediating role of visual cortical function may provide mechanistic insights into the neurobiological substrates underlying the effects of *Alistipes*-related metabolites on behavioral deficits in MDD. Indeed, numerous studies have documented visual cortical dysfunction in MDD patients as well as its relations to affective symptoms and cognitive impairments that characterize this disorder (Le et al., 2017; Lu et al., 2022; Wu, Lu, Kong, & Zhang, 2023).

Several limitations should be mentioned in this work. First, our study employed untargeted metabolomics analysis, which only permitted relative quantification of metabolites and thus precluded us from conducting group comparisons due to the confounds of batch effects. Targeted metabolomics analysis will be needed to more precisely quantify metabolites. Second, the fairly modest sample size and potential medication effect may increase the instability of the results. Future studies in a larger sample of drug-naive patients with MDD are warranted to confirm our findings. Third, the cross-sectional design does not allow any conclusion on causality. Future longitudinal or experimental studies will be required to discern between cause and effect. Fourth, some lifestyle factors, such as diet (e.g. Mediterranean diet) and physical activity, are likely to influence our results and thus require careful consideration in the future. Fifth, while mediation analysis was a key focus of our work and allowed us to identify the pathways from metabolome to behavior, we must acknowledge that mediation analysis has its own limitations. Specifically, mediation analysis, a regression-based approach, examines the relationship between variables and can suggest potential pathways, but it cannot establish the directionality of the pathways (Rijnhart et al., 2021; Tönnies, Schlesinger, Lang, & Kuss, 2023). The specification of independent and dependent variables is often based on our existing knowledge and assumptions, which may not fully capture the complexity of biological interactions. Other potential pathways, such as those from behavior to metabolome, are of great interest and merit further research in our future work. Finally, this study enrolled only Chinese participants, which may limit the generalizability of our findings.

In conclusion, using comprehensive multi-omics profiling and integrated analysis, this study provides a proof of concept that *Alistipes* and its related metabolites play a critical role in the pathophysiology of MDD via the microbiota–gut–brain axis. More broadly, our findings may inform novel therapeutic interventions targeting *Alistipes* or its effects on the brain in MDD patients.

## Supporting information

Liu et al. supplementary material 1 Liu et al. supplementary material

Liu et al. supplementary material 2 Liu et al. supplementary material

## Supplementary material

The supplementary material for this article can be found at http://doi.org/10.1017/S003329172510072X.
