# Supplementary material for: Multi-omics analyses of the gut microbiome, fecal metabolome, and multimodal brain MRI reveal the role of Alistipes and its related metabolites in major depressive disorder
Source: Psychol Med. 2025 Jul 7;55:e190. doi: 10.1017/S003329172510072X (PMC12270276; doi:10.1017/S003329172510072X)
Supplement: Liu et al. supplementary material 2 — Liu et al. supplementary material [file S003329172510072Xsup002.docx]

**­Supplementary materials**

**Table S1.** Associations between Alistipes and brain imaging in MDD patients after additionally controlling for antidepressant types, illness duration and BMI.

| **Brain imaging measure** | ***pr*** | ***P*_FDR_** |
| --- | --- | --- |
| ALFF_Occipital_Mid_R | -0.33 | 0.014 |
| ALFF_Parietal_Sup_R | -0.27 | 0.033 |
| ALFF_Parietal_Inf_R | -0.33 | 0.014 |
| ALFF_Angular_R | -0.27 | 0.033 |
| ALFF_Precuneus_L | -0.29 | 0.029 |
| ALFF_Precuneus_R | -0.28 | 0.031 |
| fALFF_Cingulum_Mid_R | -0.27 | 0.024 |
| fALFF_Cingulum_Post_L | -0.23 | 0.043 |
| fALFF_Calcarine_L | -0.23 | 0.043 |
| fALFF_Occipital_Sup_L | -0.30 | 0.013 |
| fALFF_Occipital_Mid_L | -0.31 | 0.013 |
| fALFF_Parietal_Sup_L | -0.25 | 0.036 |
| fALFF_Parietal_Inf_L | -0.26 | 0.026 |
| fALFF_Angular_L | -0.25 | 0.036 |
| fALFF_Precuneus_L | -0.35 | 0.006 |
| fALFF_Precuneus_R | -0.32 | 0.013 |
| FCD_Postcentral_R | -0.39 | 0.001 |

Abbreviations: ALFF, amplitude of low-frequency fluctuations; BMI, body mass index; fALFF, fractional amplitude of low-frequency fluctuations; FCD, functional connectivity density; FDR, false discovery rate; Inf, inferior; L, left; MDD, major depressive disorder; Mid, middle; *pr*, partial correlation coefficient; R, right; Sup, superior.


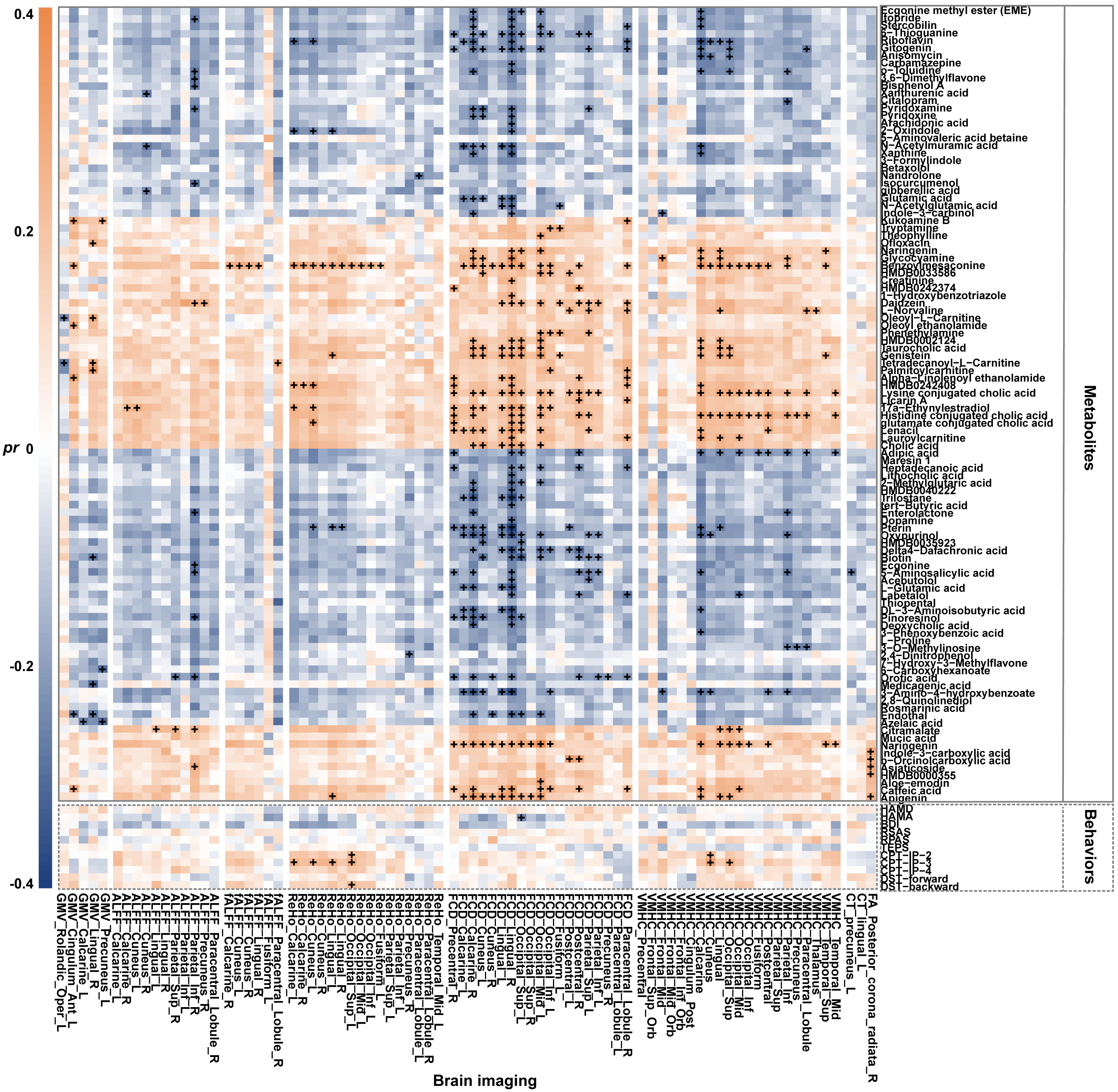


**Figure S1.** Heatmap showing the associations of brain imaging with metabolites and behaviors after additionally controlling for antidepressant types, illness duration and BMI in MDD. The color represents partial correlation coefficient. **+** *P*＜0.05, FDR corrected. Abbreviations: ALFF, amplitude of low-frequency fluctuations; Ant, anterior; BDI, Beck Depression Inventory; BMI, body mass index; CPT-IP, Continuous Performance Task-Identical Pairs; CT, cortical thickness; DST, Digital Span test; FA, fractional anisotropy; fALFF, fractional amplitude of low-frequency fluctuations; FCD, functional connectivity density; FDR, false discovery rate; GMV, gray matter volume; HAMA, Hamilton Rating Scale for Anxiety; HAMD, Hamilton Rating Scale for Depression; HC, healthy controls; Inf, inferior; L, left; MD, mean diffusivity; MDD, major depressive disorder; Mid, middle; R, right; ReHo, regional homogeneity; RPAS, Revised Physical Anhedonia Scale; RSAS, Revised Social Anhedonia Scale; TEPS, Temporal Experience of Pleasure Scale; Sup, superior; VMHC, voxel-mirrored homotopic connectivity.


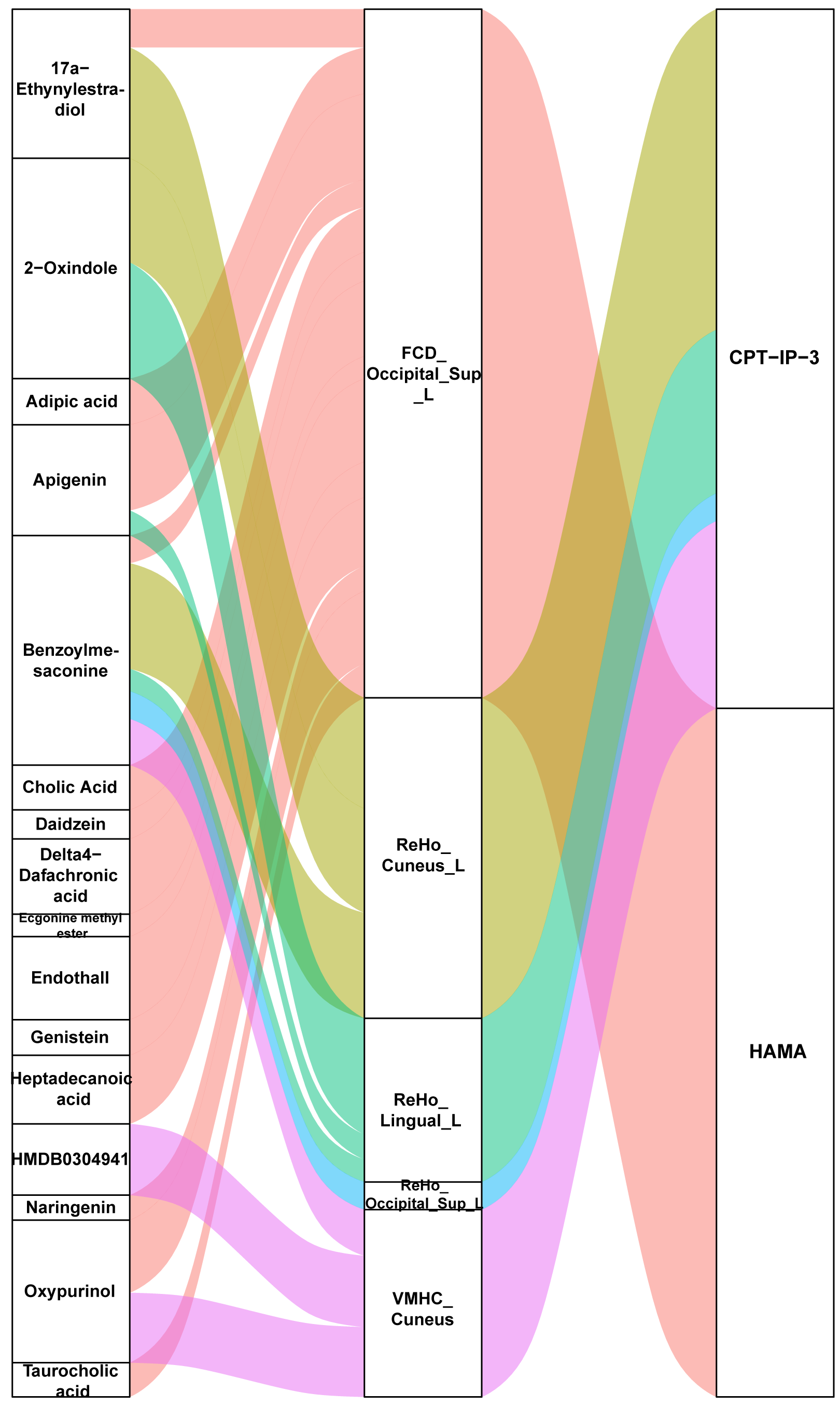


**Figure S2.** Parallel coordinates chart showing significant mediation pathways where brain functional measures (middle) mediated the associations of metabolites (left) with affective symptoms and cognition (right) in MDD patients after additionally controlling for antidepressant types, illness duration and BMI. The curved lines connecting the panels indicate the mediation effects, with colors corresponding to different brain functional measures. Abbreviations: BMI, body mass index; CPT-IP, Continuous Performance Task-Identical Pairs; FCD, functional connectivity density; HAMA, Hamilton Rating Scale for Anxiety; L, left; MDD, major depressive disorder; ReHo, regional homogeneity; Sup, superior; VMHC, voxel-mirrored homotopic connectivity.
